# Personal Values for Sustainable Eating: A Preliminary Investigation of a Value-Based Planned Behavior Model

**DOI:** 10.3390/nu17132224

**Published:** 2025-07-04

**Authors:** Edoardo Del Conte, Lucia Tecuta, Elena Tomba

**Affiliations:** Department of Psychology, University of Bologna, Viale Carlo Berti Pichat 5, 40127 Bologna, Italy; edoardo.delconte2@unibo.it (E.D.C.); lucia.tecuta2@unibo.it (L.T.)

**Keywords:** sustainability, dietary behaviors, personal values, theory of planned behavior, intention-behavior gap, pro-environmental behavior

## Abstract

**Background/Objectives:** The adoption of sustainable eating behaviors is not only crucial for environmental health but also has significant implications for individual health outcomes. A deeper understanding of the psychological determinants underlying such changes is needed. The Theory of Planned Behavior (TPB) has been widely used to understand the psychological factors influencing health behaviors, including dietary choices. Recent advances suggest integrating additional psychological constructs, such as personal values, to enhance TPB’s predictive power and the effectiveness of related behavioral interventions. **Methods:** A novel Food-Related Personal Values Questionnaire (FRPV-Q) was developed based on Schwartz’s circumplex model of basic values, and the role of these food-related personal values within an enhanced TPB framework was tested. Statistical analyses were conducted to explore the structure of the questionnaire. **Results:** Three components were identified within the FRPV-Q: Openness, Health and Security, and Autonomy (Kaiser–Meyer–Olkin test = 0.576). The regression analyses highlighted the potential role of personal values in predicting sustainable eating behaviors (adjusted R^2^ = 0.318). Specifically, an orientation toward autonomy, hedonism, and self-directionality appeared to hinder the adoption of sustainable food choices, while an orientation toward health, security, and openness to novelty was found to promote more sustainable dietary choices. **Conclusions:** The results offer preliminary insights into the role of personal values in food-related behaviors. Future research aimed at understanding and promoting pro-environmental food-related behaviors should rigorously investigate the topic. Carefully tailored value-based psychological interventions may prove beneficial for the general population in the promotion of sustainable dietary lifestyles.

## 1. Introduction

The adoption of sustainable eating behaviors is not only crucial for environmental health but also has significant implications for individual health outcomes. The research indicates that a dietary shift can prevent approximately 11 million deaths annually, primarily by reducing the incidence of diet-related chronic diseases, such as cardiovascular disease, diabetes, and certain cancers [1,2]. Despite the clear benefits, a substantial gap exists between knowledge and action in dietary behaviors among the general population [3,4], posing a challenge for healthcare providers and public health practitioners.

Among the causes that hinder its application in care settings is the failure to integrate sustainable dietary patterns and practices of international guidelines at the individual behavioral level, making them more accessible and understandable for consumers. Some researchers have recently suggested the development of Sustainable and Healthy Dietary Behaviors (SHDBs), a framework that connects sustainable dietary patterns and practices outlined in international guidelines [5,6] to sustainable eating behaviors, such as food selection, preparation, consumption, and disposal, at the level of “dietary behavior” [7]. They identified several patterns of sustainable and healthy practices, including the holistic integration of all practices (from purchasing to disposal) or the selective adoption of specific practices (e.g., avoiding plastic) to provide clearer behavioral insights for developing strategies that promote consumers’ sustainable and healthy dietary practices. Current research is rapidly advancing in the effort to effectively assess sustainable and healthy diets (e.g., [8]). However, available assessment instruments remain heterogeneous and fail to account for certain key facets of a truly sustainable and healthy diet [9].

Furthermore, in this process of change, psychology plays a key role, and a deeper understanding of the psychological determinants underlying such behavioral change is needed. The Theory of Planned Behavior (TPB) [10] has been widely utilized to understand the psychological determinants of health psychology and health behavior change [11,12], including nutrition and dietary choices. The TPB posits that behavior is directly influenced by behavioral intentions, which are in turn shaped by attitudes, subjective norms, and perceived behavioral control (PBC); intentions function as a mediator between antecedents and behaviors, and PBC is the only antecedent that directly influences both intentions and behavior. Although the TPB has shown its usefulness specifically in predicting sustainable and healthy food choices [13,14], it often falls short in fully explaining and predicting complex behaviors such as dietary habits [4,15]. Moreover, although existing TPB theory-based health behavior change interventions reliably promote health behavior change, new theoretical framework-based interventions considering additional relevant factors and pathways toward health behavior change are needed [12].

Recent advances [16,17,18,19] suggest integrating additional psychological constructs, such as personal values, to enhance TPB’s predictive power and to improve interventions aimed at health behavior change. Personal values, deeply ingrained and stable principles that guide behaviors, have been indeed shown to influence a wide range of health-related behaviors [20,21,22]. Incorporating values into the TPB model could provide a more holistic understanding of the motivations underlying sustainable and healthy eating patterns, thus offering valuable insights for designing more effective dietary interventions. In clinical practice, understanding the interplay between personal values and dietary behaviors can aid healthcare providers in developing personalized nutrition counseling strategies.

One of the most influential theoretical models of personal values is the Schwartz circumplex model of basic values [20,23], which identifies 10 basic values: Stimulation, Self-Direction, Universalism, Benevolence, Conformity, Tradition, Security, Power, Achievement, and Hedonism. These ten basic values can be grouped into four higher-order categories: Openness to Change, Self-Transcendence, Conservation, and Self-enhancement. The circumplex structure of Schwartz’s theory reflects the dynamic nature of values, their compatibilities (e.g., Universalism is theoretically compatible with Benevolence), and conflicts (e.g., Tradition is theoretically conflictual with Stimulation); the four macro-categories are also conflictual or compatible with one another, in a blurred continuum along two higher-order bipolar dimensions (Openness to Change vs. Conservation, Self-Enhancement vs. Self-Transcendence). The theory has been refined recently [24], but the new model is more complex and less influential. Schwartz’s model proved to be stable across different cultures: while value endorsement changed considerably across different cultures, the circumplex structure remained constant [25].

The circumplex model has proven to be a useful framework for studying the influence of values on Pro-Environmental Behaviors (PEBs): openness to change values seem to have a small positive effect on a broad range of PEBs, from sustainable corporate performance to activism, from recycling behavior to organic food consumption and transportation behaviors [26,27,28], while self-enhancement values such as hedonism and power have a negative effect [21,29]. In relation specifically to dietary behaviors, self-transcendence values, in particular universalism and benevolence, are correlated with higher organic food consumption [30] and less meat consumption [31], also cross-culturally [32]. A recent study conducted to investigate differences between home and restaurant settings identified a positive association between sustainable food choices and Self-Transcendence values, and a negative one between sustainable food choices and Conservation values in both contexts [33]. Ultimately, core values interact also with food-specific time perspectives: individuals endorsing Self-Transcendence value are more oriented towards long-term time perspective and tend to behave according to long-term gains, while individuals endorsing Self-Enhancement values tend to stick to short-term time perspective and short-term gratification behaviors, also concerning food choices [34].

To the best of our knowledge, however, the above empirical research exploring how personal values influence the prediction of committed intentions and behaviors related to sustainable and healthy diets according to the SHDB framework [6] lack targeted measures of food-related values. Additionally, research exploring how personal values influence the prediction of committed intentions and behaviors related to sustainable and healthy diets, especially when compared to the original well-established health behavior change variables of the TPB model, is needed. This study aims to address these gaps by developing and testing a novel measure to assess food-related personal values based on Schwartz’s circumplex model of basic values [20,23]. We argue that considering specifically food-related values related to the framework [6], instead of general, universal values (not specifically connected with food), can be of use in deepening our current understanding of determinants of dietary choices, in line with past research [35,36].

Specifically, two hypotheses were formulated (a first hypothesis pertaining to the structure of the measure and a second hypothesis concerning the role of personal values as predictors of SHDBs in the TPB model):

- The measure to assess food-related personal values will demonstrate a multidimensional structure consistent with Schwartz’s circumplex model of 10 basic values grouped in four higher-order categories (Openness to Change, Conservation, Self-Enhancement, Self-Transcendence), organized along two higher-order bipolar dimensions: Openness to Change Vs. Conservation and Self-Enhancement Vs. Self-Transcendence [20];
- Adding food-related personal values as antecedents of food choice as conceptualized by the SHDBs [6] to the TPB model will enhance its predictive capability [16,17,37]. In particular, Self-Transcendence and Openness to Change values will be positively correlated with sustainable and healthy food choices [29,30,32], and Self-Enhancement values will be negatively correlated with sustainable and healthy food choices [21,38]. Conservation values have yielded mixed results in the literature, making it challenging to formulate a linear hypothesis [29].

The literature on PEBs is striving to bridge not only the “intention–behavior gap”, but also the “value–action gap”, which is recognized as a still unresolved phenomenon in environmental research [39,40]. The research will support the development of more effective, value-based dietary assessment tools and interventions. By aligning dietary recommendations with patients’ core values, practitioners can potentially increase adherence to healthy eating plans, thereby improving clinical outcomes and ultimately contributing to a more sustainable food system.

The present study is to be considered a theory-informed exploratory effort aimed at initiating a line of research focused on the role of food-related personal values in dietary behaviors and, specifically, in pro-environmental dietary behaviors.

## 2. Materials and Methods

### 2.1. Participants and Procedure

After the approval of the Bologna University Bioethics Committee (Prot. n. 0210063, 27 July 2023), a convenience sample from the general population was recruited online using the data collection platform Qualtrics. The inclusion criteria were: (1) having reached the consent age and (2) a good command of Italian language. After signing the informed consent, adult participants filled in the following self-report questionnaires:

- An ad hoc questionnaire to collect socio-demographic information (e.g., gender, age, educational level, and occupation) and anthropometric measurements (e.g., weight and height) composed of 31 items.
- A TPB questionnaire, adapted from Gifford et al. [19], composed of 20 items on a 7-point Likert scale (the score ranged from 1 to 7); this questionnaire assesses the original TPB model’s antecedents (attitudes, social norms, PBC, and intentions) concerning sustainable and healthy dietary behaviors. The questionnaire was developed by the authors with the aim of expanding the TPB applied to sustainable dietary behaviors and, besides the original antecedents, novel constructs, such as obligation felt, were also added to the questionnaire. For the purpose of this study, only the 20 items concerning the original antecedents of the TPB were employed.
- The Sustainable and Healthy Dietary Behaviors (SHDB) scale [6] focuses on identifying sustainable and healthy dietary behaviors among consumers. The SHDB is a 30 items self-report questionnaire based on domestic and international guidelines that comprises five subscales (food choice, storing, cooking, consumption, and disposal) on a 7-point Likert scale format (the score ranged from 0 to 6). The back-translation method was used. The SHDB showed an excellent internal consistency (α = 0.93). This study focused on sustainable eating behaviors in terms of what food participants eat rather than where they buy it and how they cook it. Therefore, only the Food Choice subscale of the SHDB questionnaire was used to conduct our analyses. This scale includes items investigating the types of foods chosen (such as “I choose foods with eco-friendly packaging” and “I choose whole grain food”), but also items investigating the quantities of healthy foods (e.g., “I choose 200 g or more fruit”) and the salt amount (e.g., “I choose foods and cuisine that are not salty”).
- The Food Related Personal Values Questionnaire (FRPV-Q) is a questionnaire developed in this study to assess basic values related to dietary behaviors based on the ten values from Schwartz’s circumplex theoretical model. The questionnaire provides instructions for participants, asking them to evaluate the statements in relation to their food choices. The FRPV-Q item responses are constructed on a 7-point Likert scale format (1 to 7). The definition of each value, as well as the final items of the FRPV-Q questionnaire in both Italian and English, can be found in Table 1. For the purpose of this study, only the Italian version of the FRPV-Q was employed.

### 2.2. FRPV-Q Development

To develop the FRPV-Q, starting from the definition of the ten basic values and from the items that Schwartz and colleagues proposed in their studies [20,23,38] and instruments [41,42,43], 10 items investigating the construct in relation to food and dietary behaviors were developed. Each item was derived from Schwartz’s value definitions and contextualized to food-related decision-making through iterative refinement. For example, the definition of Hedonism refers to “pleasure and sensuous gratification” [38], and the Schwartz Value Survey [20] refers to Hedonism with the words “pleasure, enjoying life, self-indulgent”; the value was therefore reflected in food choices, focusing on the pleasure deriving from eating foods particularly aligned to taste. The final item is “If I really like a certain food, I choose it without thinking about anything else”. All the items were developed by one researcher (E.D.) and checked by two researchers (E.T. and L.T.). After receiving approval from all three researchers, the items were included in the FRPV-Q. Moreover, pilot testing with six participants was conducted to evaluate item clarity and face validity.

An a priori analysis with G-Power [44,45] showed that the recommended sample size for our regression models was 129 participants (Linear Multiple Regression, R^2^ increase, standard values); therefore, our sample size (n = 157) was adequate. The database will be made available upon request. Scores are reported using means (±std. dev) as indicator.

### 2.3. Statistical Analyses

Descriptive statistics were run to analyze socio-demographic data and psychological variables scores. To explore the structure of the FRPV-Q questionnaire, a Principal Component Analysis (PCA) with Promax rotation and Pearson’s correlations between the components resulting from the PCA were conducted. PCA was chosen over more rigorous factor analyses, such as Exploratory and Confirmatory Factor Analysis (EFA and CFA), for two main reasons: (1) the sample size was limited and arguably inadequate for a rigorous structural analysis; (2) the nature of the study was exploratory and, in line with the literature criteria, PCA allowed a less demanding preliminary testing of the instrument, also because of the lower sample size requirements compared to EFA or CFA [46,47].

**Figure 1:**
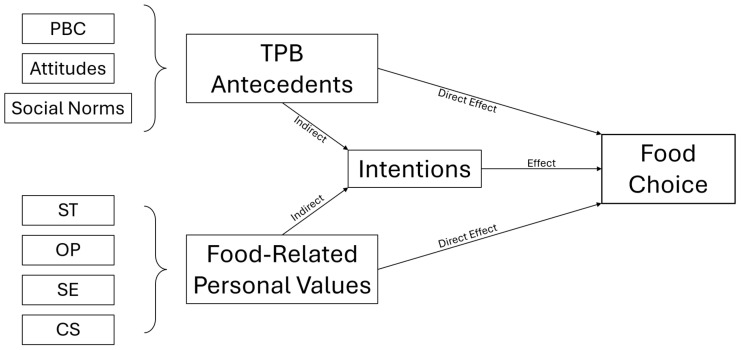
Mediation models: PBC = perceived behavioral control; ST = self-transcendence; OP = openness to change; SE = self-enhancement; CS = conservation.

To evaluate whether the inclusion of food-related personal values as antecedents can improve the predictive capability of the TPB model on SHDB-Food Choice, two steps were necessary. First, to investigate the role of intention in mediating between the antecedents (both the TPB antecedents and the FRPV-Q) and the behavior (SHDB-Food Choice); second, to evaluate the predictive capabilities of the models, with or without the mediation component from intentions. Subsequentially, two mediation models were analyzed (see Figure 1) with the antecedents as predictors (TPB antecedents in the first model, FRPV-Q in the second one), the TPB intentions as a mediator, and the SHDB-Food Choice as the independent variable.

**Figure 2:**
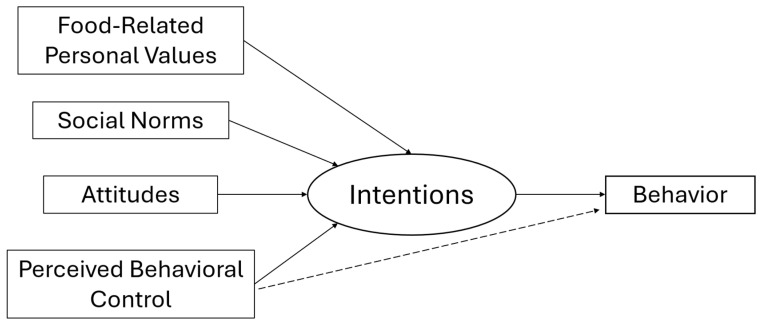
Hypothesized value-based TPB (V-TPB) model.

To compare the predictive capability of the original TPB model and of an extended TPB model including food-related values (V-TPB, see Figure 2), two separate linear regression analyses were conducted: one employing only the original TPB antecedents (attitudes, social norms, and PBC), and a second one the V-TPB, adding FRPV-Q components as additional antecedents to the original TPB model. A stepwise methodology was used to compare adjusted R^2^ scores to identify the more predictive model. All the analyses were conducted using the software JASP, version 0.18 [48].

## 3. Results

### 3.1. Sample Characteristics

A total of 193 individuals took part in the study, but only 157 (81%) individuals completed all the questionnaires. All the participants were Italian, and most of them were female (73.2%). Almost the totality of the sample reported a medium (60%) or a low income (28%), whilst only 11% reported medium-high income and 0.5% reported high income. The age of participants varied from 18 to 69 years, with a mean age of 30.8; the reported BMIs varied from 16.4 to 41.3, with a mean BMI of 22.9.

The mean score of the subscale “Food Choice” of the SHDB questionnaire was 3.82. In the TPB questionnaire, the Attitudes towards sustainable eating consumption were very high, with a mean score of 6.08; PBC followed, with a mean score of 5.46; Intentions showed a mean score of 4.04. Social Norms showed the lowest score in the TPB questionnaire with a mean of 3.12. Descriptive statistics for the variables can be found in Table 2.

### 3.2. Exploring the Structure of the FRPV-Q

The PCA yielded three principal components of the FRPV-Q, composed of three, four, and two items for a total of nine final items. The tenth item, Achievement, was excluded due to equal (but opposite) loading onto two separate components; for more detailed information, see Table 2. The structure of FRPV-Q differs from Schwartz’s circumplex model of 10 values grouped into four principal components from which the values’ definitions were extrapolated and related to food. The structure was relatively solid, with a 54% explained variance and a significant (*p* < 0.001) chi-squared test. The Kaiser–Meyer–Olkin (KMO) test was also acceptable (overall Measure of Sample Adequacy = 0.572), although values < 0.80 are generally considered optimal.

The first component was named Openness (OP) and gathered Stimulation, Universalism, and Conformity values. OP featured positive loadings from the Stimulation and Universalism values and negative loading from the Conformity value (see Table 3). These values, according to Schwartz’s circumplex model, belong to different categories (Stimulation belongs to Openness to Change, Universalism to Self-transcendence, and Conformity to Conservation). The second FRPV-Q component was named Health and Security (HS) and included Security, Benevolence, Power, and Tradition values with positive loadings from all four items (Table 3); these values are adjacent in the original circumplex model but also belong to separate categories (Benevolence to Self-transcendence, Security and Tradition to Conservation, Power to Self-enhancement). The third FRPV-Q component was named Autonomy (AU) and featured positive loadings from Hedonism and Self-direction values (Table 3); Hedonism, according to Schwartz’s model, belongs both to the Self-enhancement and to the Openness to Change categories, while Self-direction only belongs to Openness to Change. A single-factor solution was not considered as it was not deemed appropriate for the construct in exam.

Descriptive statistics and Pearson’s correlation between the components identified through the PCA were conducted. The highest scoring component of the FRPV-Q was Openness, followed by Autonomy and Health and Security; the correlation analyses showed that the three components were uncorrelated (Table 4).

### 3.3. Comparing Theoretical Models

Two mediation analyses were conducted to investigate the mediating role of intentions in the relationship between antecedents and sustainable eating behaviors (see Figure 1). The first mediation analysis, which considered intentions as a mediator between the original TPB antecedents (Attitudes, Social Norms, and PBC) and the Food Choice subscale of the SHDB questionnaire, yielded a non-significant indirect effect of the TPB antecedents on self-reported Food Choice (0.065 < *p* < 0.387). The second mediation analysis replicated the model, with the addition of the three components of the FRPV-Q emerging as significant from the PCA (OP, HS, and AU) and obtaining similar results (0.059 < *p* < 0.996). The direct and total effects of both TPB and FRPV-Q antecedents on Food Choice-SHDB were instead significant, particularly concerning PBC, the OP component, and the HS component (*p* < 0.001). More detailed information can be found in Tables S1 and S2. The direct effects of intentions on the Food Choice subscale of the SHDB questionnaire were investigated as well, revealing a significant model with an explained variance of about 8% (R^2^ = 0.084, *p* < 0.001, β = 0.289).

Based on such results, two separate regression analyses using stepwise methodology were subsequentially conducted without considering the mediating effect of TPB intentions to compare the two theoretical models (TPB and V-TPB) in the prediction of Food Choice. The first regression analysis included only the TPB antecedents (attitudes, PBC, social norms), and the second one included both the TPB antecedents and the three components of the FRPV-Q (V-TPB model) as predictors, and both analyses featured the Food Choice subscale of the SHDB questionnaire as the dependent variable.

The first stepwise regression analysis showed significant effects (*p* < 0.001) of PBC on SHDB-Food Choice (see Table S3). Social norms and attitudes were excluded from the model due to the insignificance of the predictive effect; PBC alone explained 19% of the variability in SHDB-Food Choice (R^2^ = 0.187). The second stepwise regression analysis yielded four possible significant models, with an adjusted R^2^ ranging from 0.182 (PBC alone) to 0.318 (PBC with the three FRPV-Q components). The significant increase in the adjusted R^2^ suggested that the models with better fit were model 4 (included predictors: PBC, OP, and HS) and model 5 (included predictors: PBC, OP, HS, and AU). Please see Table 5 for additional information.

In Model 4, all included predictors showed a positive significant predictive effect (OP: β = 0.233, *p* = 0.002; PBC: β = 0.299, *p* < 0.001; HS: β = 0.338, *p* < 0.001) on the SHDB-Food Choice subscale. In model 5, AU showed a small, negative, mildly significant predictive effect (β = −0.137; *p* = 0.049) on SHDB-Food Choice, and all the other predictors showed a positive significant predictive effect (OP: β = 0.225, *p* = 0.002; PBC: β = 0.315, *p* < 0.001; HS: β = 0.346, *p* < 0.001). Despite model 4 having fewer variables in the model, the explained variance of model 4 (adjusted R^2^ = 0.304) was comparable to model 5 (adjusted R^2^ = 0.318).

## 4. Discussion

To the best of our knowledge, this is the first empirical study that presents the development of a short and broad measure (FRPV-Q) to evaluate food-related personal values towards sustainable and healthy diets and explores the interplay between such values towards sustainable and healthy dietary behaviors within an enhanced TPB framework. Although the FRPV-Q is based on the Schwartz’s circumplex model, our analysis identified nine values in relation to only three distinct components—OP, HS, and AU—each comprising values that, in Schwartz’s model, belong to different sections of the model. This unexpected result could be attributable to the use of PCA, which could have constrained the emergence of a structure consistent with the circumplex model.

The first FRPV-Q component (OP) includes three items reflecting Stimulation and Universalism (which conceptualize diversity positively as an interest in novel experiences and a belief in diversity as an asset) and Conformity (which conceptualizes diversity negatively, as reluctance to choose uncommon foods). People with high scores in the OP component of the questionnaire are theoretically more inclined to explore novel foods, new culinary techniques and technologies, and in general, more inclined to experiment with dietary changes. The second FRPV-Q component (HS) includes the four values of Security, Benevolence, Power, and Tradition in relation to food (see Table 1). People with high scores in the HS component are theoretically more inclined to stick to their current dietary habits, but also to care about the healthiness of food, food workers, and the environment. The third FRPV-Q component (AU) captures a self-reliant approach to food choices, based on the Hedonism and Self-direction values (see Table 1). People with higher scores in the AU component of the questionnaire are theoretically more inclined to choose their diets based on their personal knowledge and taste.

Although the measure to assess food-related personal values developed in this paper did not reflect Schwartz’s circumplex model, and our first hypothesis was not confirmed, the FRPV-Q still possesses significant predictive power regarding self-reported sustainable dietary behaviors, which highlights its practical value and relevance in understanding and predicting them. The resulting structure is preliminary and exploratory, as the use of PCA does not allow a rigorous validation of the scale. The observed three-component structure does not imply a rejection of the Schwartz’s model but may reflect an empirical simplification driven by the analytic procedure. It is also important to consider that there is no guarantee that the same structure and results will be obtained in other populations: personal values are by nature culturally shaped, and it is possible that the set of values that were identified as predictors of SHDBs in this paper are different in other cultures. Moreover, all the participants in our sample were Italian speakers and, therefore, only the Italian version of the questionnaire was employed; results in other languages could vary.

### 4.1. Integration of the FRPV-Q into the TPB Model

The results support our second hypothesis about the utility of the integration of additional psychological variables within the original TPB model. In line with previous studies [17,49,50], adding personal values improves the predictive power of the original TPB model in relation to more sustainable food choices. More specifically, the three value components of FRPV-Q (OP, HS, and AU) added as antecedents provide an increase in the ability of the extended theoretical model (V-TPB) compared to the original TPB model to predict self-reported food choices. Prior research [51] using the classic TPB model has explained 20–40% of the variance in individual behaviors. In our study, the TPB model alone explained a lower bound of this range (approximately 19%), which remains consistent with the existing literature. However, the V-TPB model, incorporating FRPV-Q values, increased the explained variance to approximately 32%, supporting the added predictive strength. All three FRPV-Q components were significant predictors of the SHDB-Food Choice subscale. Moreover, the three components were not correlated to each other, and their independence on self-reported sustainable food behaviors is therefore assumed.

### 4.2. Interpreting the FRPV-Q Components and Their Impact on Sustainable Food Choices

Participants with higher OP scores (encompassing values of Stimulation and Universalism and Conformity even though the latter in a negative loading) at the FRPV-Q tended to report more sustainable food choices, meaning that a value orientation towards the exploration of novelty favors sustainable food consumption. Previous research identified a role of the openness personality trait in contrasting food neophobia, which is associated with a poorer dietary quality [52,53] and, in Italy specifically, with the refusal of ethnic food [54]. Although values and personality are independent predictors of behaviors [55], these results are aligned. Moreover, the OP component features Universalism and Stimulation values, which have been consistently associated with PEBs [29,30,32]. The Conformity value has a negative loading on the OP component and is to be therefore considered as a reverse item; conformity values have been shown to have a negative correlation with PEBs; therefore, this result is also aligned with the literature [29].

Participants with higher HS scores (encompassing Security, Benevolence, Power, and Tradition values about food) also tended to adopt more sustainable food choices, meaning that people who care more about the health and security of themselves and others are more likely to purchase and consume sustainable foods. Although this finding is apparently in contrast with the existing literature, in which self-enhancement values (such as Power) and conservation values (such as Tradition) are negatively correlated with PEBs [29], some consideration can be made. Firstly, it is important to note the specific context in which the analysis was conducted: Italy has a strong connection with the Mediterranean diet and traditional dishes are often sustainable [56], which might explain why the Tradition value seems to favor sustainable food choices. Moreover, the Power value item focuses on the price of foods, which may reflect a concern for quality rather than for status [57], meaning people with a high score in this item favor high-quality foods compared to industrial and processed foods, which are less sustainable. Notably, our Power item focuses on the social status and prestige component of the value rather than on the dominance and control component; the facet we considered might be closer to the Conservation category than it is to the Self-Enhancement category. Our questionnaire includes in the HS component also Benevolence and Security (self-transcendence and conservation values, respectively), which are contextually connected to the greater frequency of self-reported PEBs [29]. Although belonging to separate categories, the four values included in the HS component are adjacent in the original circumplex model.

Lastly, participants with higher AU scores (encompassing Hedonism and Self-direction values about food) tended to adopt less sustainability-oriented dietary behaviors, reflecting their orientation towards self-reliance; similar effects have been detected in other studies [26,58]. Zhu and colleagues [59] found that higher scores in the Autonomy scale of the Psychological Well-Being scales are associated with poorer dietary outcomes in obese patients, and although psychological well-being and values are not overlapping constructs, these results converge on the fact that higher self-reliance and autonomy can lead to more difficulties in changing one’s dietary habits. Previous research also focused on the role of Hedonism, the other item included in the AU component of the questionnaire, highlighting it as a strong barrier to healthier behavioral changes; results from this study support that argument [21,29]. The nature of the interrelations between the three components is, similarly to the observed structure, exploratory.

In sum, our hypotheses are only partly confirmed, due to the different structure of the FRPV-Q compared to Schwartz’s circumplex model. In particular, the hypotheses concerned a positive association between self-transcendence and openness to change values and sustainable dietary behaviors and a negative association between self-enhancement values and sustainable dietary behaviors. The OP component is positively associated with SHDB-Food Choices and featured positive loadings from both openness to change and self-transcendence values, and this result is in line with the hypotheses. The HS component is also positively associated with SHDB-Food Choice and, in line with our hypotheses, featured positive loadings from self-transcendence values; however, the HS component also featured a positive loading from a value pertaining to self-enhancement (Power), which is unexpected, as discussed above. The AU component is negatively associated with SHDB-Food Choices and featured positive loading from Hedonism, which is in line with the hypotheses, but also from Self-Direction, which pertains to the openness to change values and would theoretically favorably impact PEBs; as discussed above, this result can be due to the wording of the FRPV-Q and to its broadness.

### 4.3. The Role of Intentions and of the Other TPB Antecedents

In our sample, intentions to eat sustainable and healthy food did not mediate the effect of antecedents (both original TPB factors and food-related values) on self-reported food choices. This contradicts classical TPB research, which posits a mediating role of intentions [10,60]. While for antecedents such as attitudes and HS values the indirect effect of Intentions was close to significance—suggesting that a larger sample might yield significant results—the other variables had *p*-values far from the significance threshold. A body of research [61,62] suggests that the role of intentions in predicting behaviors is sub-optimal or marginal, and that most behaviors are influenced by subtle cues or non-conscious processes, and the results of these analyses are consistent with this body of research.

Finally, despite not being included in our hypotheses, our results concerning the other original TPB antecedents confirm the available literature. Specifically, PBC has a prominent role in predicting both actual and self-reported behavior [63] and is the only antecedent directly linked to behavior [10]; attitudes alone cannot explain behavior, especially in the field of PEBs, in line with the “attitude–behavior gap” literature [64,65,66]; social norms have several times been identified as the weakest predictor of behaviors, also specifically in PEB research [64,67].

### 4.4. Practical Implications

The intention–behavior gap is a serious issue in sustainability research [39,68] and health psychology [11], and it should be addressed also by investigating complex models that may help to predict eating behaviors and support clinical practice both in terms of assessment and intervention options. Our results are preliminary, and this study represents an exploratory effort—and an invitation for researchers—to delve into the study of food-related personal values and their possible role in promoting PEBs. In light of these considerations, some practical implications can be drawn.

According to our results, future assessment strategies and intervention efforts aimed at modifying and improving sustainable and healthy dietary behaviors should consider food-related personal values. The current literature offers a wide variety of instruments to assess personal values, which can be distinguished from one-another based on the level of abstraction, the broadness, and the response format [69]. The measures available for studying personal values are valid and reliable, but also long and time-consuming; many researchers [41,42,43] have developed shorter questionnaires to address this issue. A short self-report measure to assess food-related personal values has been developed in this study aiding to better identify areas of change toward healthier and more sustainable dietary lifestyles: the assessment of lifestyle is indeed underutilized, despite considerable evidence of its effectiveness in both clinical and general populations [70]. By leveraging patients’ core values, practitioners (such as dietitians and nutritionists) can potentially increase adherence to healthy and sustainable eating plans, thereby improving clinical outcomes. By aligning dietary recommendations with patients’ core values, practitioners can potentially increase adherence to healthy eating plans when they encapsulate openness to experiences and concern for the health and security of foods. Inversely, patients’ orientation towards autonomy, hedonism, and self-directionality values should be considered detrimental to the adoption of sustainable food choices and to dietary change. Additionally, our work builds on the research of Kawasaki and colleagues [6] by contributing to the development of a consumer profile that considers not only demographic characteristics but also food-related values. Developing accurate consumers’ profiles while also considering value orientations can be of use both in clinical practice and in guiding sustainability promotion campaigns and helping policymakers, facilitating the adherence to the 2030 Agenda for Sustainable Development [71] of all the stakeholders involved.

From a practical standpoint, future interventions promoting eudaimonic instead of hedonic positive experiences in relation to sustainable dietary lifestyles can be of help. Ryan and Deci [72] have summarized research on psychological well-being as falling into two general groups: the hedonic viewpoint focuses on subjective well-being, happiness, pain avoidance, and life satisfaction [73,74,75], whereas the eudaimonic viewpoint focuses on meaning and self-realization and defines well-being in terms of the degree to which a person is fully functioning [76] or as a set of wellness variables such as self-actualization and vitality [72]. Eudaimonia is also defined as “the subjective positive experiences […] in which actions are fully engaged, reflectively endorsed, and aligned with deeply held values and beliefs” [77]. In the field of clinical psychology, a widely used approach that incorporates value-based theories is Acceptance and Commitment Therapy (ACT) [78], which promotes engagement in behavioral changes by focusing on the link between personal values and committed actions not only to reduce psychological distress, but to also develop a new relationship with one’s experiences and to improve eudemonic psychological well-being [79,80].

Although ACT was initially developed to target mental health issues, behavioral change has always been its additional relevant target: of the first three studies conducted on ACT one was on diet and another on tolerance of physical pain [81]. In recent years, the interest in ACT applied to health behavioral change has grown, also specifically for eating behaviors and weight control and several studies highlight its effectiveness [82,83]. A recent scoping review by Cox et al. [84] highlighted that ACT might be beneficial for modifying eating behaviors in adolescents with obesity or overweight; additionally, a recent metanalysis [85] found that ACT had a significant, albeit small, effect on weight loss on overweight patients, suggesting that a tailored ACT intervention could be an effective tool for modifying eating behaviors.

ACT has recently been proposed as a promising approach to support the development of PEBs while also mitigating climate-change-related distress [22]. The flexible and versatile theoretical framework and intervention techniques of ACT can be of importance in understanding and improving facilitators and decreasing barriers to sustainable development and to an ecological transition. For example, according to Hayes and colleagues [78,86], the concept of experiential avoidance is crucial in explaining maladaptive behaviors and could be intertwined with the “collective denial of climate and ecological decline” [22]. Moreover, ACT is also linked to mindfulness, which is one of the most promising factors in promoting sustainability [87,88] also in relation to healthy and sustainable eating choices.

## 5. Limitations and Conclusions

The present study has some limitations that need to be discussed. Firstly, the sample size was relatively small for the mediation analyses, and our results should be interpreted with caution. In addition, the current sample size may compromise the stability and replicability of the extracted component structure; therefore, results pertaining to a three-components structure of food-related values should be interpreted with caution

As previously noted, our findings do not invalidate Schwartz’s circumplex structure but may be an empirical simplification ascribable to the use of PCA; further validation and replication efforts are required before considering the FRPV-Q a standard evaluation method for personal values in the food domain. Future research should employ larger and more diverse cross-cultural samples with more robust techniques like EFA and CFA, both of which are better suited to testing circumplex structures, to assess whether the theoretical model is preserved when items are adapted to the food context in different cultures. Although the use of PCA can be considered a limitation, it was deemed appropriate because of the explorative nature of the present study, consistently with the available literature [46,47]. Moreover, the different wording used in our questionnaire to investigate values might have introduced ambiguity or shifted the underlying constructs being measured.

A second limitation concerns the assessment tools used in our study. During the development of the FRPV-Q, a formal content validity assessment was not conducted. Furthermore, the TPB questionnaire used taken from Gifford et al. [19] did not allow us to directly evaluate the behavior and, consequently, may not account for some facets of intentions that could have predicted or mediated the relationship between antecedents and behavior more strongly and consistently. It is equally possible that mediation analysis using other constructs as independent variables (e.g., green purchase behavior) rather than a food choice subscale could provide different results. More targeted research is needed to establish with a greater degree of certainty if intentions really are negligible in predicting and promoting sustainable food choices. A similar concern can be expressed about the role of attitudes that was negligible in our study; however, a more in-depth assessment of attitudes (both the explicit and the implicit components) could yield different results. Additionally, a more accurate assessment of attitudes could enable an analysis of the interplay between values, attitudes, and food choices.

Moreover, it is important to note that, whilst shorter versions of values inventories have the advantage of being more convenient and easier to administer, this may come at the cost of lower reliability [69]. The AU component is composed of only 2 items, and this could represent a limitation; it would perhaps be appropriate to employ 2 items per area instead of 1, totaling the questionnaire at 20 items, for the purpose of higher reliability. Moreover, our items were worded differently compared to most of the currently available value questionnaires: first-person sentences have been employed, with which participants could agree or disagree, depicting behaviors or situations that reflect Schwartz’s values (for a similar approach, see [66]). This formulation could have caused the PCA to not reflect the circumplex model but rather to identify other underlying patterns of personal values. Further analyses employing a more diverse and larger sample, paired with EFA and CFA, should be conducted in future research. The divergent and convergent validity of the questionnaire should also be addressed. Moreover, whilst a single-factor solution and a total score for the questionnaire was not deemed appropriate for the construct in exam (due to the fact that identical score in a value questionnaire could represent opposite value orientation), future studies could consider it if the factorial analysis supports it.

In addition, self-reported behavior and actual behavior often differ from each other. A longitudinal design with a measure of actual dietary behaviors, perhaps with novel technologies [89,90] such as ecological momentary assessment, can provide data that are more adherent to consumers’ true behavioral patterns.

In conclusion, focusing specifically on food-related personal values instead of general, universal values could be an effective way to deepen our understanding of determinants underlying food choices. One possible future line of research consists in verifying if food-related values can predict food choice with more accuracy than universal values, to clarify whether food-related personal values are useful or redundant and unnecessary. Additional data are needed to confirm this hypothesis, but the preliminary results are promising.

## Figures and Tables

**Table 1:** Definition of Schwartz’s values and 10 items of the food-related personal values questionnaire in English and Italian.

Value	Value Definition	Item (English)	Item (Italian)
**Stimulation** *(* *Openness to Change)*	Excitement, novelty, and challenge in life	I like to try novel foods and new culinary experiences, even if they are unusual.	Mi piace provare nuovi cibi e nuove esperienze culinarie, anche inusuali
**Hedonism** *(* *Openness to Change/Self-Enhancement)*	Pleasure and sensuous gratification for oneself	If I really like a food, I chose it without thinking about anything else.	Se un alimento mi piace particolarmente, lo scelgo senza curarmi di nient’altro
**Self-Direction** *(Openness to Change)*	Independent thought and action—choosing, creating, and exploring	When I choose what to eat, I don’t care about others’ opinions on certain foods.	Quando scelgo cosa mangiare, non mi curo molto dell’opinione comune su determinati alimenti
**Universalism** *(* *Self-Transcendence)*	Understanding, appreciation, tolerance, and protection for the welfare of all people and of nature	To me, diversity, also culinary, is an asset.	Per me la diversità, anche culinaria, è una ricchezza
**Benevolence** *(* *Self-Transcendence)*	Preservation and enhancement of the welfare of people with whom one is in frequent personal contact	When choosing a certain food, I find it of the utmost importance that the supply chain cares about workers, consumers, environment, and animals.	Nella scelta di un alimento, per me è fondamentale che la filiera produttiva si prenda cura allo stesso modo di lavoratori, consumatori, ambiente e animali
**Tradition** *(* *Conservation)*	Respect, commitment, and acceptance of the customs and ideas that traditional culture or religion provides	Respecting culinary traditions is more important than experimenting.	Rispettare le tradizioni e le usanze alimentari è più importante che sperimentare
**Conformity** *(* *Conservation)*	The restraint of actions, inclinations, and impulses that are likely to upset or harm others and violate social expectations or norms.	I don’t like to choose unusual or uncommon foods.	Non mi piace scegliere alimenti inusuali o non comuni
**Security** *(* *Conservation)*	Safety, harmony, and stability of society, relationships, and self	When choosing a food, I pay absolute attention to how much the food is available and safe for my health.	Nella scelta di un alimento faccio estrema attenzione a quanto l’alimento è disponibile e sicuro per la mia salute
**Power** *(* *Self-Enhancement)*	Social status and prestige, control, or dominance over people and resources	I don’t like to buy cheap foods, I prefer expensive, luxury foods.	Non apprezzo acquistare cibi economici ma solo alimenti costosi e “di lusso”
**Achievement** *(* *Self-Enhancement)*	Personal success through demonstrating competence according to social standards	I choose a food instead of another mainly based on my goals (e.g., managing body weight and shape, impressing others).	Scelgo un alimento piuttosto che un altro principalmente sulla base dei miei obiettivi (ad es. gestire peso e forma, fare impressione sugli altri)

**Table 2:** Descriptive statistics.

	Food Choice ^1^	Attitudes	Norms	PBC ^2^	Intentions
**Mean**	3.82	6.05	3.12	5.46	4.04
**Std. Dev.**	0.82	0.90	1.06	1.16	0.70

^1^ Food Choice refers to the Food Choice subscale of the SHDB; ^2^ PBC = Perceived Behavioral Control.

**Table 3:** Component loadings in the principal component Analysis.

**Item**	**Component 1:** **Openness (OP)**	**Component 2:** **Health and Security (HS)**	**Component 3:** **Autonomy (AU)**
**Stimulation** *(Openness to Change)*	0.845		
**Universalism** *(Self-Transcendence)*	0.810		
**Conformity** *(Conservation)*	−0.778		
**Security** *(Conservation)*		0.828	
**Benevolence** *(Self-Transcendence)*		0.645	
**Power** *(Self-Enhancement)*		0.414	
**Tradition** *(Conservation)*		0.407	
**Hedonism** *(Openness to Change/Self-Enhancement)*			0.748
**Self-direction** *(Openness to Change)*			0.725
**Achievement** *(Self-Enhancement)*		0.435	−0.420

**Table 4:** Descriptive statistics and Pearson’s correlation between the three components resulting from the PCA.

	Openness	Health and Security	Autonomy
**Openness**	-		
**Health and Security**	r = −0.108; *p* = 0.179	-	
**Autonomy**	r = −0.038; *p* = 0.632	r = 0.098; *p* = 0.224	-
**Mean**	5.78	3.73	4.41
**Std. Dev.**	1.16	0.97	1.31

**Table 5:** Regression analysis including FR-PV and TPB antecedents as predictors of the SHDB-Food Choice subscale.

Model Summary	R	R^2^	Adjusted R^2^		
**Model 4: PBC, HS, and OP**	0.564	0.318	0.304		
**Model 5: PBC, HS, OP, and AU**	0.580	0.337	0.318		
**Model**	**Variable**	**Std. Error**	**β**	**t**	***p***
**Model 4**	Intercept	0.409		1.563	0.120
	PBC	0.053	0.299	4.050	<0.001
	HS	0.061	0.338	4.677	<0.001
	OP	0.050	0.233	3.213	0.002
**Model 5**	Intercept	0.436		2.203	0.029
	PBC	0.052	0.315	4.279	<0.001
	HS	0.061	0.346	4.827	<0.001
	OP	0.050	0.225	3.130	0.002
	AU	0.043	−0.137	−1.986	0.049

PBC = Perceived Behavioral Control; HS = Health and Security; OP = Openness; AU = Autonomy. Social Norms and Attitudes were considered but are not included due to the stepwise methodology.

## Data Availability

All the data retrieved for the current study will be made available upon reasonable request. For any further information, please contact the corresponding author Elena Tomba.

## Supplementary Materials

The following supporting information can be downloaded at: https://www.mdpi.com/article/10.3390/nu17132224/s1. Table S1: Mediation analysis with intentions as mediating between TPB antecedents and the food choice subscale of the SHDB; Table S2: Mediation analysis with intentions mediating between FRPV-Q components and the food choice subscale of the SHDB; Table S3: Regression analysis including TPB antecedents as predictors of SHDB-Food Choice subscale. Social norms and attitudes were considered but are not included due to stepwise methodology.
