# Supplementary material for: Personal Values for Sustainable Eating: A Preliminary Investigation of a Value-Based Planned Behavior Model
Source: Nutrients. 2025 Jul 4;17(13):2224. doi: 10.3390/nu17132224 (PMC12252425; doi:10.3390/nu17132224)
Supplement: Supplementary file 1 [file nutrients-17-02224-s001.zip › nutrients-3679712-supplementary.pdf]

Personal Values for Sustainable Eating: A Preliminary Investigation of a Value-Based Planned Behavior Model

Table S1: Mediation analysis with Intentions mediating between TPB antecedents and the Food Choice subscale of the SHDB.

Direct effects	*Estimate*	*Std. Error*	*z-value*	*p-value*
**Attitudes**	0.020	0.081	0.247	0.805
**Social Norms**	-0.016	0.059	-0.272	0.786
**Perceived Behavioral Control**	0.266	0.058	4.552	< 0.001
Indirect effects	*Estimate*	*Std. Error*	*z-value*	*p-value*
**Attitudes**	0.043	0.023	1.843	0.065
**Social Norms**	0.012	0.014	0.864	0.387
**Perceived Behavioral Control**	0.022	0.014	1.551	0.121
Total effects	*Estimate*	*Std. Error*	*z-value*	*p-value*
**Attitudes**	0.063	0.078	0.804	0.421
**Social Norms**	-0.004	0.059	-0.069	0.945
**Perceived Behavioral Control**	0.288	0.056	5.107	< 0.001

Table S2: Mediation analysis with Intentions mediating between FRPV-Q components and the Food Choice subscale of the SHDB.

Direct effects	*Estimate*	*Std. Error*	*z-value*	*p-value*
**Openness**	0.200	0.046	4.316	< 0.001
**Health & Security**	0.325	0.064	5.099	< 0.001
**Autonomy**	-0.065	0.046	-1.424	0.154
Indirect effects	*Estimate*	*Std. Error*	*z-value*	*p-value*
**Openness**	0.016	0.011	1.499	0.134
**Health & Security**	0.037	0.019	1.886	0.059
**Autonomy**	0.000	0.008	-0.005	0.996
Total effects	*Estimate*	*Std. Error*	*z-value*	*p-value*
**Openness**	0.216	0.046	4.660	< 0.001
**Health & Security**	0.361	0.060	6.056	< 0.001
**Autonomy**	-0.066	0.047	-1.391	0.164

Table S3: Regression Analysis including TPB antecedents as predictors of the SHDB Food Choice subscale. Social Norms and Attitudes were considered but are not included due to Stepwise methodology.

**Model summary**	**R**		**R^2**	**Adjusted R^2**
Perceived Behavioral Control	0.433		0.187	0.182
**Variable**	Std. Error	β	t	p
Intercept	0.299		7.127	<.001
Perceived Behavioral Control	0.053	0.433	5.783	<.001
